# The Challenge Non-Typhoidal *Salmonella* (CHANTS) Consortium: Development of a non-typhoidal *Salmonella* controlled human infection model: Report from a consultation group workshop, 05 July 2022, London, UK

**DOI:** 10.12688/wellcomeopenres.19012.2

**Published:** 2023-09-21

**Authors:** Christopher Smith, Emma Smith, Christopher Chiu, Jay Hinton, Blanca Perez Sepulveda, Melita Gordon, Robert K.M. Choy, Peter W.S. Hill, James E. Meiring, Thomas C. Darton, Megan E. Carey, Graham Cooke, Malick M. Gibani, CHANTS Consortium

**Affiliations:** 1 Department of Infectious Disease, Imperial College London, London, W2 1PG, UK; 2 Institute of Infection, Veterinary and Ecological Sciences, University of Liverpool, Liverpool, L69 3BX, UK; 3 Malawi-Liverpool-Wellcome Trust Clinical Research Programme, Blantyre, Malawi; 4 PATH, Seattle, Washington, 98121, USA; 5 Department of Infection, Immunity and Cardiovascular Disease, University of Sheffield, Sheffield, S10 2TN, UK; 6 Department of Medicine, University of Cambridge, Cambridge, UK

**Keywords:** Salmonella; Salmonella Typhimurium; Non-Typhoidal Salmonella; Human Challenge Model; Controlled Human Infection Model; Vaccines

## Abstract

Invasive non-typhoidal *Salmonella* disease (iNTS) is a major cause of morbidity and mortality globally, particularly as a cause of bloodstream infection in children and immunocompromised adults in sub-Saharan Africa. Vaccines to prevent non-typhoidal *Salmonella* (NTS) would represent a valuable public health tool in this setting to avert cases and prevent expansion of antimicrobial resistance. Several NTS and combination typhoidal-NTS vaccine candidates are in early-stage development, although the pathway to licensure is unclear due to challenges in conducting large phase III field trials.

Controlled human infection models (CHIM) present an opportunity to accelerate vaccine development for a range of enteric pathogens. Several recent typhoidal *Salmonella* CHIMs have been conducted safely and have played pivotal roles in progressing vaccine candidates to pre-qualification and licensure. The Challenge Non-Typhoidal *Salmonella* (CHANTS) consortium has been formed with funding from the Wellcome Trust, to deliver the first NTS CHIM, which can act as a platform for future vaccine evaluation.

This paper reports the conclusions of a consultation group workshop convened with key stakeholders. The aims of this meeting were to: (1) define the rationale for an NTS CHIM (2) map the NTS vaccine pipeline (3) refine study design and (4) establish potential future use cases.

## Disclaimer

The views expressed in this article are those of the author(s). Publication in Wellcome Open Research does not imply endorsement by Wellcome.

## Introduction

Controlled human infection models (CHIM) provide a valuable method to study infectious diseases ^1^. Studies involving the controlled exposure of healthy volunteers to pathogenic or attenuated pathogens have been used for several decades to better understand the pathophysiology of disease – complementing insights from pre-clinical and field studies. Historically, a major use of such models has been in the field of vaccine development, in which a CHIM can be used to generate an early indication of vaccine efficacy and to suggest immune correlates of protection ^2^. This is particularly true for enteric pathogens for which, to date, CHIM studies have had a pivotal role in accelerating vaccine development for pathogens such as *Vibrio cholerae* and *Salmonella* Typhi, and are predicted to have an important role in vaccine development for *Shigella spp* ^3– 5^.

There is growing interest and awareness by regulatory agencies of the utility of CHIM studies in vaccine development for diseases which occur sporadically, or for which field efficacy trials are difficult to conduct. One such disease is invasive non-typhoidal *Salmonella* (iNTS). Over 2600 non-typhoidal Salmonella (NTS) serovars exist, which affect a broad host-range and are associated with a spectrum of clinical phenotypes in human infection, exhibiting significant geographic variation ^6^. In high-income settings, NTS infections primarily cause a self-limiting enterocolitis, termed diarrhoeal NTS (dNTS). In contrast, invasive non-typhoidal *Salmonella* disease (iNTS) typically presents as a non-specific febrile illness – often without gastrointestinal symptoms – following invasion into sterile sites causing bloodstream infection, meningitis, or sepsis ^7^. iNTS disease is a major cause of morbidity and mortality in sub-Saharan Africa (SSA), primarily affecting immunocompromised hosts., The most recent GBD 2019 data estimate iNTS were responsible for 535,000 cases, 77,500 deaths, and 4·26 million lost disability-adjusted life years (DALYs) in 2017 ^8^. The precise burden of disease is, however, relatively poorly understood and estimates vary depending on case-definitions and methods of surveillance ^9, 10^. The global burden of diarrhoeal disease attributable to non-typhoidal *Salmonella* serovars (dNTS) is less well characterised, but likely is responsible for a larger case load – preliminary estimates from the Institute of Health Metrics and Evaluation (IHME) give estimates of 73.9 million cases and 61,600 deaths attributable to dNTS in 2019 ^11^. The World Health Organisation (WHO) has recently listed *Salmonella enterica* as a priority pathogen, identified as one of 12 families of bacteria that pose the greatest risk to human health through rising antimicrobial resistance ^12^.

Vaccines for iNTS would represent a valuable public-health tool in sub-Saharan Africa, in part because other effective methods for disease control are – arguably – slow and cost prohibitive in many endemic countries in the short-term. Several candidate vaccines are in development, including conjugate vaccines, live-attenuated oral vaccines, and those using the generalised modules for membrane antigens (GMMA) platform, typically in multivalent formulations ^13, 14^. Vaccine development for iNTS is hampered, in part, by an incomplete understanding of mechanisms and determinants of immunity during natural infection, as well as a lack of commercial incentive. In addition, the epidemiology of iNTS disease dictates that the large-scale phase III trials of vaccine candidates needed to establish efficacy will require a large financial and time commitment. The most efficient pathway to vaccine licensure and deployment is unclear.

Unlike other enteric pathogens, there is relatively limited experience of controlled human infection studies of non-typhoidal *Salmonella* serovars ^15– 17^. The use-case for an NTS CHIM in vaccine assessment is controversial ^18, 19^. Recently, the Wellcome Trust has funded the establishment of the Challenge Non-Typhoidal *Salmonella* (CHANTS) consortium, to develop a first-in-human model of *Salmonella* Typhimurium infection using contemporary strains. This consortium brings together stakeholders with expertise in *Salmonella* biology and controlled human infection studies to design a safe clinical study that de-risks the development of an NTS CHIM. The vision of this program is to develop an iNTS CHIM that dovetails with future iNTS vaccine efficacy studies, whilst simultaneously addressing fundamental questions on the immunological basis of susceptibility to iNTS disease.

A new CHIM for NTS will only significantly advance vaccine development if careful consideration is given to study design at an early stage, particularly with regards to selection of clinically meaningful endpoints and setting appropriate efficacy thresholds. Additionally, understanding the needs of key stakeholders is necessary to map out a potential use case in a vaccine product development pathway.

To address these issues, a consultation group workshop meeting was convened on 5 ^th^ July 2022, with the aim of gathering input into the design and use-case of the proposed NTS CHIM. The consultation group meeting aimed to link the CHANTS consortium with key stakeholders in the NTS vaccine field, including regulators, academic researchers, funders, and vaccine developers. This meeting report summarises the key discussion points and outcomes from the workshop. We begin by reviewing the rationale for the development of an NTS CHIM and identify key learning points from other human challenge models for enteric diseases. This report focuses on priority topics discussed in the meeting, including challenge strain selection; choice of endpoints; clinical considerations and mapping out a future use-case.

## Rationale for an iNTS controlled human infection model

Unlike human host-restricted *Salmonella* Typhi, NTS serovars typically have a broad host range ^20, 21^. Several animal models have been developed to assess NTS candidate vaccines ^22^. Many of these models have provided valuable pre-clinical insights into host-pathogen interactions but do not always replicate the manifestations of disease in a context relevant for vaccine development. Historically, human challenge with wild-type or attenuated NTS serovars has been performed in a small number of volunteers, but a large-scale programme of activities for vaccine assessment has not previously been established ^18^.

In the absence of an established NTS CHIM, there are several unknowns that hamper vaccine development. An NTS CHIM provides opportunities to understand NTS disease pathogenesis (both diarrhoeal and invasive) and the mechanisms that drive these distinct clinical phenotypes. Investigation of immune responses to the challenge agent at time points before and after exposure will provide insights into mechanisms of protective immunity and identification of immune correlates of protection. Once established, an NTS CHIM would provide a platform to evaluate vaccine efficacy and support licensure of vaccine candidates currently in early phase evaluation. Furthermore, controlled challenge with NTS can elucidate novel target antigens for future vaccine development, identify diagnostic biomarkers, and facilitate assessment of the impact of infection on the host gastrointestinal microbiome. 

Whilst the anticipated benefits of conducting an NTS CHIM are many, there are several unknowns in relation to clinical responses to challenge. In particular, an NTS CHIM in healthy, immunocompetent adults from high-income settings is unlikely to accurately replicate an invasive disease phenotype most frequently observed in immunocompromised children with risk factors, including prematurity, malnutrition, and malaria infection, in lower- and middle-income countries (LMICs). 

These uncertainties have been discussed at several expert meetings in the preceding years and have been discussed elsewhere ^18, 19^. Acknowledging the gaps which remain in this field, it was concluded that to address these questions, a CHIM could be established to characterise the response to wild type NTS challenge in order to understand if – and how – a human challenge model of NTS disease could be used for future vaccine assessment.

The CHANTS consortium has since been funded by Wellcome Trust, including key partners from Imperial College London, PATH, University of Oxford, Malawi-Liverpool-Wellcome Trust Clinical Research Programme, and the University of Liverpool. This consortium aims to establish an NTS CHIM that offers maximum insight from controlled human infection, whilst being underpinned by the central principle of volunteer safety.

### NTS human challenge – safety and ethics

In the first session, the workshop discussed the overarching considerations pertaining to the safety and ethical acceptability of an NTS challenge model. The principal tension in the development of an NTS CHIM is developing a framework that prioritises safety and ethical considerations, whilst simultaneously providing a clinically meaningful way to test vaccines that protect against invasive disease. Specifically, the development of a dNTS challenge model would pose fewer safety and ethical challenges but would not necessarily help to accelerate vaccines designed to address iNTS disease.

A CHIM that reliably achieves bloodstream infection (BSI) would have the greatest utility when viewed solely from the perspective of vaccine efficacy assessments against invasive disease. Previous *Salmonella* Typhi and Paratyphi CHIMs are good examples of this, as BSI occurs following challenge with these serovars in healthy, immunocompetent adults and responds rapidly to effective antibiotic treatment ^23, 24^. In contrast, BSI with *Salmonella enterica* subspecies *enterica* serovars Typhimurium and Enteritidis is almost exclusively observed in clinically vulnerable populations with impaired host immunity ^7, 25^. This includes a high mortality rate (5–15%) and the potential to seed deep-seated infections. It is plausible that silent or pauci-symptomatic BSI (as observed in typhoidal *Salmonella* challenge models) could be seen after challenge of healthy volunteers with NTS, but this is unlikely to represent the same pathophysiological processes as those associated with iNTS disease in the target population for vaccination.

Well recognised host-risk factors associated with invasive disease includes extremes of age, recent malaria infection, sickle cell disease, malnutrition, advanced HIV infection in adults and other defects in the immune system. Volunteers with these characteristics could arguably never be safely or ethically challenged with virulent *Salmonella* ^7, 25^. Most CHIMs typically start by challenging exceptionally healthy volunteers in a narrow age range (e.g., healthy UK volunteers aged 18–50). It was acknowledged outright this population are not representative of the population at greatest risk of iNTS and are unlikely to develop invasive disease.

The relative contribution of pathogen specific factors in achieving invasive disease within the context of the model was discussed by the consultation group and is summarised below (Study Design). Importantly, it was noted that *Salmonella* Typhimurium ST313 isolates have been found in both invasive and diarrhoeal infections. 

The consortium had a high degree of confidence in the establishment of a colonisation and gastrointestinal NTS disease model in immunocompetent adults. It was acknowledged that the prospect of achieving an invasive disease model representative of iNTS is less secure. The session finished by highlighting the key safety considerations which would be adopted to de-risk the model for participants, namely: establishment of an independent data and safety monitoring committee; thresholds for initiation of antimicrobial therapy; quality assurance of strain manufacture and administration, and close liaison with public health authorities including the local health protection team and the UK Health Security Agency (UKHSA) in view of potential secondary household or community transmission.

### NTS vaccine landscape

As listed WHO priority pathogens, invasive NTS isolates are increasingly associated with multi-drug resistance, significantly limiting treatment options ^26– 29^. Development of effective vaccines for NTS may contribute to reduced case numbers, thus reducing antibiotic use and the expansion of antimicrobial resistance ^30^. The next session aimed to put an NTS CHIM within the context of vaccines currently under assessment. Key questions to address included where a CHIM would sit within the vaccine product development pathway, and what additional data could be generated that would meaningfully accelerate vaccine development.

In this workshop, Professor Cal MacLennan outlined the NTS vaccine landscape as of July 2022. He defined several key considerations in NTS vaccine development. These included:

•    Whether an NTS vaccine could be developed as a stand-alone product or formulated in combination with other serovars

•    Whether such a vaccine could provide protection against both invasive and diarrhoeal disease

•    What would be the most appropriate age of administration and alignment within the Expanded Programme on Immunisation (EPI) schedule?

•    What is the expected duration and putative mechanisms of protection?

•    What would be the efficacy of a vaccine in individuals with comorbidities at higher risk of developing NTS?

Several NTS vaccines are currently under development which aim to target *S*. Typhimurium and *S*. Enteritidis. The most advanced include a bivalent conjugate vaccine comprising core and O-polysaccharides conjugated to serovar specific phase I flagellin protein (FliC) (University of Maryland/Bharat Biotech), where Phase I studies are complete ^31^. Additionally, a bivalent vaccine developed by GSK Vaccines for Global Health (GVGH), based on the generalised modules for membrane antigens (GMMA) platform, is approaching Phase I ^32^. Several other vaccines are in pre-clinical testing including a bivalent NTS vaccine developed by Boston Children’s Hospital based on the multiple antigen presenting system (MAPS) technology ^13^.

Several expanded-valency formulations of NTS and typhoidal *Salmonella* vaccines candidates are also in pre-clinical testing. These include a trivalent NTS (O-antigen conjugated to flagellin for Typhimurium and Enteritidis combined with Vi-TT Typbar-TCV [Centre for Vaccine Development, University of Maryland/ Bharat Biotech]); a trivalent NTS + Vi-CRM197 (GVGH); and a trivalent NTS + Vi-DT (IVI/SK). In addition, hypothetical quadrivalent vaccines for enteric fever ( *S*. Typhi and Paratyphi A) with *Salmonella* Typhimurium and Enteritidis have been proposed ^13^.

Analyses indicate that iNTS vaccines would be cost-effective in resource-limited countries, particularly when combined with other *Salmonella* serovars, as the priority for protection is a time-limited period where the burden of disease is highest – specifically the first three years of life. Cost effectiveness would rely on having the capacity to mass produce and roll-out a vaccine at low-cost ^33^.

As phase I data from vaccine trials become available, it was acknowledged there is a need to engage with vaccine developers early, particularly with respect to where a CHIM would fit in an already well-defined development pipeline. Until readouts from a pilot NTS CHIM are known, the future utility of the model for vaccine assessment remain uncertain.

## Study design

Having considered the rationale for an NTS CHIM and its potential utility for supporting vaccine development, the consultation group reviewed different proposed challenge study designs, based on a draft template protocol. 

The originally proposed study was to perform oral challenge with two wild-type strains of *Salmonella* Typhimurium administered in sodium bicarbonate in a dose escalation model targeting an attack rate of 60–75%.

### Challenge strain selection

This study proposes to use two challenge strains belonging to ST19 and ST313 sequence types. This approach would directly address the contribution of African ST313 lineages to systemic disease compared with non-invasive isolates.

Dr Blanca Perez Sepulveda (University of Liverpool) outlined the rationale for the choice of strains used in this model. All human challenge models are limited in the number of strains that can be used and are frequently criticised for failing to replicate the global diversity of strains responsible for infection in endemic settings. Where possible, bacterial strains used for challenge should: be representative of circulating strains; be sensitive to multiple antibiotics used for treatment; have a traceable history from isolation; have been well characterised phenotypically and genotypically *in vitro* and *in vivo*; be capable of causing the disease of interest; and possess a representative array of antigens and virulence factors.

The NTS serovars commonly isolated in BSI varies by location, but usually include *Salmonella* serovars Typhimurium and Enteritidis ^34^. *S.* Typhimurium accounts for approximately two-thirds of iNTS in Africa. Sequencing of isolates from across Africa has identified multilocus sequence type (MLST) 313 as the major cause of invasive disease, which is distinct from the sequence types 19 and 34, which are more frequently associated with gastrointestinal disease in high-income settings ^35^. ST313 appears to have undergone sequential evolutionary changes that converge towards a genotype associated with invasive disease, in the same way as reported for *Salmonella* Typhi and Paratyphi serovars ^26, 36^. The key changes are characterized by stepwise genomic degradation typified by multiple gene deletions and pseudogene formation, often in genes associated with gastrointestinal survival ^26^. The data suggests that ST313 has adapted to a human niche and can evade mucosal immunity ^36– 38^. Similar findings have been observed in invasive *S*. Enteritidis ST11 isolates from Africa ^35^. Transmission of invasive isolates is complex and likely involves a combination of zoonotic reservoirs (particularly for *S*. Enteritidis) as well as human-to-human transmission ^20, 21^. Invasive NTS isolates are increasingly associated with multi-drug resistance, significantly limiting treatment options ^26– 29^.

This study proposes to manufacture challenge stocks from the ST313 lineage 2 reference strain D23580, which can be considered as an archetypal isolate representative of strains causing invasive disease in sub-Saharan Africa. D23580 was originally isolated in 2004 from a blood culture taken from a 24-month-old HIV-negative child at Queen Elizabeth Central Hospital, Blantyre, Malawi. D23580 harbours four plasmids, including pSLT-BT, which encodes drug resistance to chloramphenicol, ampicillin, streptomycin, sulphonamides, and trimethoprim. The presence of drug resistant mobile genetic elements within D23580 was felt not to represent a significant risk when balanced against the benefits of using a well-characterised strain that is reliably sensitive to fluoroquinolones and more representative of isolates detected from endemic settings. In parallel, it was proposed to manufacture a challenge stock of the ST19 4/74 strain, originally isolated from a calf in England in 1974. Genomic analysis indicates that it is an archetypal ST19 strain, representative of current clinical infections. Both strains have undergone detailed genotypic, transcriptomic, proteomic, and phenotypic characterisation ^39^. Consensus was reached that challenge with highly characterised pathogens is advantageous from a participant safety perspective. 

These proposals are predicated on the hypothesis that challenge of healthy volunteers with ST313 will be associated with a distinct clinical phenotype, as compared to challenge with ST19. There is a substantial body of phenotypic, genomic, and epidemiological evidence to indicate that infection with specific African lineages of ST313 will be associated with less gastrointestinal disease as compared with ST19 pathovariants ^35^. Whilst challenge with *S*. Enteritidis would be valuable, resource limitations currently preclude stock development, and there was agreement that the most valuable data, within resource constraints, would be generated from a *S.* Typhimurium CHIM.

The consultation group were invited to consider challenge dosing. It was raised that the dose needed to achieve 60–75% attack rates might vary between strains and depend on what clinical endpoints are selected. For example, a relatively low dose of ST19 may cause a higher proportion of volunteers developing diarrhoeal disease compared with ST313. In view of the novelty of this model, it was suggested that a starting dose of 10 ^3 ^CFU was rational, based upon prior experience with *S.* Typhi and Paratyphi A challenge administered with sodium bicarbonate ^23, 24^.

All strains will be manufactured to GMP standard at the pilot biomanufacturing facility of the Walter Reed Army Institute of Research (MD, USA), following protocols previously established for typhoidal *Salmonella* studies. It was recommended that stability testing should be performed at least annually, and more frequently at the start of the study to ensure the challenge agent remains stable. Characterisation tests should include antibiotic susceptibility testing, whole genome sequencing and growth characteristics in different media and survival tests as a minimum. 

### Study endpoints

The selection of appropriate endpoints is critical to ensure utility of the model in future vaccine efficacy studies. An NTS CHIM could be designed to result in colonisation, diarrhoeal disease, systemic illness and/or bacteraemia. It was emphasised that these endpoints aren’t mutually exclusive and that a participant may progress from one state to another. Exposure to the gastrointestinal tract is a prerequisite for the development of invasive disease, but kinetics of invasion after exposure are poorly understood. The consultation group discussed the relative merits of developing a colonisation/early infection model, or a disease model. It was acknowledged that the primary goal of vaccine development is to protect against invasive disease, but that protection against diarrhoeagenic disease would also be beneficial.

The consultation group was invited to consider the following possible endpoint definitions of Salmonellosis, outlined in Table 1.

**Table 1.** Possible study endpoints to be used as composite or co-primary endpoints.

**Possible Primary Endpoints**
• Isolation of *Salmonella* Typhimurium from stool on ≥2 occasions ≥24 hours from challenge **and/or**
• Severe diarrhoea * **and/or**
• Moderate diarrhoea ** plus:
○ Fever ≥38°C on ≥1 occasion **and/or**
○ ≥1 Grade 2 systemic symptoms (abdominal pain, nausea, vomiting, tenesmus) **and/or**
• *Salmonella* Typhimurium bloodstream infection **and/or**
• Fever ≥38°C on ≥2 occasions ≥12 hours apart

*Severe diarrhoea defined as any one of (i) ≥6 loose/liquid stools in 24h (ii) >800g loose/liquid stool in 24h (iii) ≥2 stools containing gross blood in 24h **Moderate diarrhoea defined as any one of (i) 4–5 loose/liquid stools in 24h (ii) 400-800g loose/liquid stool in 24h

The consultation group discussed the merits and demerits of defining a composite endpoint or co-primary endpoints for each definition, generating attack rates for each of the definitions. Secondary and exploratory endpoints would also be generated, including duration/magnitude of diarrhoea, shedding, seroconversion, symptom profile and blood culture clearance.

Colonisation was thought to be insufficient as a surrogate marker for invasive disease. The primary concern was that colonisation as a primary endpoint will likely be achieved at a low infecting dose, preventing further dose escalation. Bloodstream infection and/or fever would be favourable in terms of model utility; however, care would be needed to minimise risks, as outlined above. It was acknowledged that the dose required to achieve invasive infection in healthy participants may be many orders of magnitude higher than in natural exposure, setting an unachievable threshold to demonstrate vaccine efficacy. The consultation group agreed that endpoints should have some flexibility, as the outcome following challenge is unknown.

There was consensus that a composite endpoint comprising colonisation, diarrhoeal disease and bacteraemia would not be informative. Co-primary endpoints would be preferable, to provide attack rates for colonisation, gastroenteritis and systemic disease and highlight strain differences. The challenge doses used could be escalated until safety signals require the escalation to be halted. Diarrhoea without fever could be designated as a secondary/exploratory endpoint so this would not preclude further dose escalation. The group highlighted the risk of prematurely ending the study if the target attack rate is achieved using a primary endpoint that is not representative of iNTS disease. There was broad consensus in having the scope to dose escalate with appropriate oversight by a data safety monitoring committee (DSMC).

Dose ranges and ceilings were also discussed. A stage might be reached where the investigators and DSMC consider the challenge dose to be grossly unrepresentative of natural exposure – at which point, consideration should be given to terminating the study. The consortium agreed that double-blind randomisation of participants is of value to maintain integrity in assessment of key subjective symptomatic outcome measures.

## Clinical considerations

In the final session related to study design, the consortium was invited to consider practical clinical aspects of study delivery. Key considerations included location, inpatient vs. outpatient model; inclusion and exclusion criteria (with safety as priority); pre-enrolment tests; infection control considerations; strategies to mitigate complicated disease and treatment protocols.

Key clinical risks and risk mitigation strategies that should inform study design are highlighted in Table 2.

**Table 2.** Risk assessment for *Salmonella* Typhimurium oral challenge. Participants should be counselled for these risks in the participant information sheet and during screening. Biological safety and laboratory risk-assessments are not detailed. The following risks were highlighted as areas of focus during protocol development but are non-exhaustive.

Possible study risks	Comments	Risk mitigation strategies
**Clinical Considerations**		
***Primary complications of Salmonella infection***		
Severe gastroenteritis with dehydration	• Typical symptoms include watery diarrhoea, bloody diarrhoea, abdominal pain, nausea, vomiting, headache, and fever • Overall duration of symptoms typically ranges from 4 to 7 days • Total duration of illness is estimated to range between 3 to 19 days ^40^.	• In-patient quarantine with continuous oversight from study physician and nurses (initial 7 days) • Daily stool output monitoring and culture • Daily assessment of symptoms and severity grading • Severe diarrhoea will be trigger antibiotic treatment • Oral rehydration for diarrhoea • Intravenous fluid replacement for severe hypovolaemia
Severe sepsis	• Complications of invasive *Salmonella* infection can include septicaemia, hypotension, tachycardia • Occur almost exclusively in clinically vulnerable and/or those who do not receive appropriate antibiotic treatment. • Estimated case-fatality rate 15-20% of iNTS in vulnerable patients ^9, 43^. • ~5% of enteric infections with non-typhoidal *Salmonella* are thought to result in bacteraemia. • May increase to ~10% depending on underlying host risk factors and serotype ^6, 44^. • Progression from enteric to systemic infection may occur more frequently in the context of altered gastric acid pH ^45^, altered microbiota (including prior antibiotic treatment) ^46^ and concomitant rotavirus infection ^47– 51^.	• Exclusion of participants at higher risk of developing severe sepsis/ invasive disease • In-patient quarantine with continuous oversight from study physician and nurses. • Daily blood cultures to detect bloodstream infection • Early antimicrobial therapy in event of bloodstream infection or clinical signs/symptoms of developing sepsis. • Backup admission to medical inpatient unit and/or high-dependency unit and/or intensive care unit if required.
Deep-seated focal infection	• Bloodstream infection can lead to extra-intestinal focal infections at any site, including aortitis, osteomyelitis, meningitis, or arthritis. • Almost exclusively in clinically vulnerable and/or those who do not receive appropriate antibiotic treatment • Sickle cell disease is a recognised risk factor for osteomyelitis • Endovascular disease is associated with atherosclerotic disease, valvular disease, or endovascular prosthesis • Meningitis is a rare, but can occur in neonates, infants <12years of and in the context of advanced HIV infection.	• Exclusion of participants at risk e.g. underlying valvular heart disease, bone/joint disease (including prosthesis, metalwork), aneurysmal arterial disease • Clinical examination to exclude valvular heart disease. • Abdominal ultrasound during screening to detect asymptomatic biliary disease and aneurysmal disease of the abdominal aorta
***Post-infectious/Para-infectious complications***		
Prolonged *Salmonella* shedding and/or microbiological relapse	• Convalescent shedding of *Salmonella* is common after symptomatic or asymptomatic NTS infection. • Microbiological relapse (with or without clinical symptoms) may develop post recovery and/or treatment • The precise duration of shedding is poorly understood. • In some instances, antibiotic treatment is associated with prolonged symptoms or higher rate of microbiological relapse. • Chronic carriage (shedding for >12months) is more common with host immunosuppression; in young children <5 and/or or in the context of biliary tract abnormalities. • Theoretical risk of secondary transmission if hygiene measures are not followed.	• Where required, use antibiotic classes not associated with prolonged shedding (e.g. fluoroquinolones) • Describe pattern of shedding by longitudinal collection of stool samples for detection of *Salmonella* spp. by PCR and culture. ○ Distinguish between PCR positive and culture positive samples. • Clearance stool cultures will be obtained upon completion of antibiotic therapy. ○ Follow guidance for typhoidal *Salmonella* i.e. three negative clearance stools. • Reinforce hygiene precautions after challenge. • Participants with chronic carriage (stool culture positive >4 weeks after antibiotics) can be referred to an Infectious Diseases specialist.
Clinical relapse	• Clinical relapse may develop post recovery and/or treatment • More common in immunocompromised patients. ○ Advanced HIV infection ^52^ ○ Chronic granulomatous disease ^53^ ○ Defects in specific cytokine pathways ^10, 54^ ○ Haematological malignancies ^55^	• Exclusion of at-risk individuals • Follow up of participants for up to 1 year • Study team contactable 24/7 ○ Participants advised to contact study team in event of new symptoms
Reactive arthritis	• Gastrointestinal and genitourinary infections are commonly recognised triggers, including with *Salmonella* spp. • Typical symptoms - mono-arthritis or oligo-arthritis, often affecting the lower limbs, axial symptoms, enthesitis and/or dactylitis. • Symptom duration is highly variable – ○ most patients will have little-to-no symptoms at 6–12 months post onset. ○ A small proportion of patients may develop symptoms lasting >12 months ^56^. • Prevalence of HLA-B27 increased in patients with reactive arthritis, although the estimates vary depending on illness definition and study design ^56– 58^. • Presence of HLA-B27 is not essential for the development of reactive arthritis.	• HLA-B27 screening prior to enrolment • Participants who develop symptoms of reactive arthritis will be referred to a Rheumatology service
Post-infectious irritable bowel syndrome (IBS)	• Post-infectious irritable bowel syndrome is estimated to occur in 3–10% of patients following bacterial diarrhoea. • Symptoms generally resolve within 1 year ^59– 61^.	• Exclusion of participants with pre-existing IBS • IBS questionnaire at baseline and post-challenge
Psychological impact of challenge and quarantine	• Study fatigue may occur due to the intense nature of the study procedures, especially during the quarantine period. • Participants may become anxious, lonely, or depressed by being confined to the quarantine unit for the minimum of 7 days.	• Anxiety and depression questionnaire during screening and after challenge. • Facilities to make phone and video calls with friends and family. • A small number of visitors may be allowed but would have to comply with a strict hygiene protocol.
***Risks associated with antibiotics***		
Direct adverse effect of antibiotics	• Antibiotic intolerance: ○ gastro-intestinal upset, nausea, vomiting or other unspecified symptoms. • Antibiotic hypersensitivity ○ Rash, angio-oedema, difficulty in breathing anaphylaxis • Abnormal liver function tests or other biochemical abnormalities • Risk of antibiotic associated diarrhoea and/or *Clostridioides* * difficile* infection.	• Baseline ECG (QTC) • Regular measurements of liver function tests and electrolyte monitoring • Keep antibiotic treatment duration as short as possible • Exclude participants with known antibiotic hypersensitivity or allergy to either of the first-, second- and third-line antibiotics (ciprofloxacin, azithromycin, or other macrolide antibiotics and cephalosporins)
Carriage of multi-drug resistant organisms (MDRO)	• Antibiotic treatment may in theory increase the risk of carriage of drug resistant bacteria.	• Stool culture for carriage of multidrug resistant organisms (Carbapenem resistant organisms and Extended spectrum beta lactamase producing *Enterobacterales*)
**Infection control considerations**		
Nosocomial spread of *Salmonella* between volunteers	• Theoretical risk of cross-transmission between volunteers impacting study endpoints	• Strict enteric precautions • Isolation of participants in side-rooms
Nosocomial spread of other pathogens, including SARS-CoV-2 (in-patient model)	• Theoretical risk of outbreak of SARS-CoV-2 (or other respiratory viruses) within quarantine facility.	• Use of personal protective equipment • Participants and investigators to have completed at least a primary SARS- CoV-2 vaccination course • Negative SARS-CoV-2 lateral flow test prior to admission to quarantine • Hospital infection prevention and control guidelines to be maintained throughout the quarantine phase • Investigators with relevant respiratory symptoms to isolate and avoid contact with volunteers.
Transmission to study investigators	• Theoretical risk of cross-transmission from study volunteers to study investigators	• Investigators will adhere to strict hygiene measures • Use of personal protective equipment per hospital guidelines for enteric infections
**Public health considerations**		
Onward community transmission (ranging from household secondary cases to outbreak)	• Person-to-person spread has been described, especially when patients are symptomatic with diarrhoea. • Transmission within households can occur if the individual excreting S. Typhimurium fails to practice effective hand washing after defecation and is subsequently involved in uncooked food preparation. • Person-to-person spread has been described, especially when patients are symptomatic with diarrhoea. • Clearance cultures following NTS infection are not typically indicated according to UKHSA guidance ^42^. ○ This contrasts with UK public health guidance following infection with the typhoidal *Salmonella* serovars ( *S*. Typhi and Paratyphi)	• In-patient quarantine during putative infectious period. • Defined de-isolation criteria ○ Complete resolution of diarrhoea (Bristol stool type 6-7) for 48 hours • Participant counselling on stringent hygiene measures until microbiological clearance confirmed • Exclusion of participation from high-risk occupational groups ○ food handlers ○ ongoing contact with highly susceptible persons or young children ○ healthcare workers • Access to screening for household and other close contacts • Inform health protection services at time of challenge and time of confirmed clearance. • Engage with national reference laboratories to sequence isolates from participants to compare with any future community outbreaks.

### Antibiotic treatment

The consultation group discussed antibiotic treatment at length. Opinions differed on the value of offering antibiotic treatment to all participants regardless of symptoms. All members agreed that antibiotic treatment would be indicated in cases of invasive disease. It was acknowledged that *Salmonella* gastroenteritis is typically self-limiting in healthy immunocompetent adults and that antibiotic treatment is not always indicated in this patient group – in part due to proposed risk of prolonged faecal shedding coupled with antibiotic related adverse events ^40, 41^. Prolonged faecal shedding is less likely when fluoroquinolones are used for treatment of sensitive strains, when compared with other classes of antimicrobials ^40^. To protect participant safety and to minimise risk of prematurely terminating infection, treatment criteria should be distinct from diagnostic criteria. Signs of bloodstream infection or persistent fever will result in immediate antibiotic treatment, as will a combination of gastrointestinal symptoms that exceed a pre-defined threshold. Asymptomatic individuals who establish colonisation (positive stool culture detected on >2 occasions) could also receive antibiotic therapy at day 14. Whilst the use of antimicrobial therapy for uncomplicated NTS infection is contentious, the consortium agreed that antibiotic treatment could be justified within the context of a volunteer CHIM study with close post-challenge monitoring and assessment of clearance cultures. The consultation group also agreed that the study information booklet and screening procedures should counsel participants of the risks and benefits of antibiotic treatment.

### Study location

Whilst extensive safety data are available from prior *Salmonella* CHIMs conducted as out-patient studies, the balance of risks, in conjunction with the novelty of challenge with NTS, led the group to favour an in-patient model. This followed from in-depth discussion regarding quarantine, balancing participant safety/public health with over-burdensome restrictions and the risk to mental well-being of participants. Discharge criteria and infection control considerations were developed with current public health guidance in mind. The UKHSA considers people non-infectious 48 hours after diarrhoea has ceased and clearance stool samples are not routinely required for NTS ^42^. In this study, participants will be quarantined for at least seven days, until resolution of diarrhoea for at least 48 hours, negative blood clearance cultures if bacteraemia develops, and be medically fit as assessed by a study clinician.

### Screening tests

Screening and pre-enrolment testing will consider inclusion and exclusion criteria to mitigate risk of adverse outcomes. Only healthy participants will be considered for participation, with targeted investigations undertaken to exclude occult conditions which may predispose to complications (biliary disease, abdominal aortic aneurysm). We will also exclude individuals with existing mild/sub-clinical irritable bowel syndrome (IBS) to avoid long-term complications post-infection. 

Serological pre-screening for prior *Salmonella* exposure was not viewed as being feasible, owing to lack of standardised assays at the time of writing. Instead, a post-challenge analysis could be stratified by baseline sero-status to inform future studies. Practically, a clinical history and/or microbiological evidence of documented *Salmonella* infection and/or prior Typhoid oral Ty21a vaccination, would exclude participation due to potential cross-reactivity.

## Future use case

The final workshop session focussed on defining the future use case for an NTS CHIM. The consultation group agreed that any model would preferably have a clear use in NTS vaccine assessment whilst simultaneously allowing investigators a platform to better understand NTS infection biology. It was acknowledged that these are not mutually exclusive aims but that trade-offs may be required given feasibility and budgetary constraints. 

A primary application of CHIMs is to define immune correlates of infection- or vaccine-derived immunity. Prior CHIMs for enteric pathogens have played a key role in elucidating serum anti-LPS IgG and vibriocidal antibodies as correlates of protection against *Shigella* spp. and *Vibrio cholerae*, respectively ^62, 63^. Whilst this study offers the opportunity to investigate a wide range of immune responses to infection, the consortium agreed certain assays should be prioritised, and standardised, to align with those targeted by NTS vaccine candidates currently in development. The study proposes to characterise antigen-specific and functional antibody responses at baseline, during acute disease, and during convalescence. O-antigen ELISA and serum bactericidal assays will be utilised to characterise the humoral response to NTS challenge. Saliva and stool samples will be collected to characterise mucosal IgA responses at baseline and following challenge, whilst peripheral blood mononuclear cells will be used to measure antibody-secreting-cell and memory B-cell activity (via ELISpot assays). High throughput functional antibody assays, biophysical antibody profiling and Fc-glycosylation studies could also be undertaken as part of so-called systems serology platforms ^64– 66^.

It was acknowledged that correlates of protection may differ for colonisation, gastroenteritis, and invasive disease. Careful consideration is needed to balance de-risking phase 3 studies by providing early efficacy signals, and undermining confidence in vaccine candidates based on surrogate endpoints that might not be relevant to invasive disease. It was voiced that identification of correlates should be hypothesis driven and follow a well-constructed sample collection schedule. Exploratory measurements will include whole blood and/or single cell transcriptomics, which could also help to identify the establishment of invasive disease in study participants.

The target population for any future NTS vaccine is anticipated to be children <5 years of age, although other groups including adults living with HIV may also benefit. Invasive disease is strongly linked to immune susceptibility; either in very young infants who lack protective antibody responses, or children with recent malaria infection, who may have macrophage and/or complement deficiencies. It was agreed that the main role of a CHIM for vaccine development would be increasing confidence that a candidate vaccine would work in these susceptible populations. Whilst a challenge study involving these groups could be considered ethically unjustifiable, it may be informative to have adult participants with recent malaria infection in an NTS challenge study and compare the data to immunogenicity studies from children administered a vaccine candidate as part of a field trial. From discussions held about the model described, there were no immediate concerns or additional issues perceived about future transfer of an NTS CHIM to endemic settings.

## Conclusions

The CHANTS workshop has successfully generated key recommendations to inform the establishment of an NTS challenge model. The aim of this workshop was to provide an expert consultation platform at an early stage of trial design. It is hoped that these contributions increase the likelihood that an NTS CHIM can be appropriately used to accelerate, and not hinder, vaccine development. Future consultation groups will likely be convened to report on preliminary data and refine the future use case of the model. Inputs from the expert consultation group will be considered alongside the outcome of public engagement and involvement meetings to inform the final trial protocol, which will be published once all approvals are in place. Continuing engagement with stakeholders will be key to defining and prioritising appropriate immunological endpoints to measure, specifically to ensure these are appropriately aligned and calibrated with assays currently being applied in iNTS vaccine trials.

Our vision is to develop an iNTS CHIM programme that dovetails with future iNTS vaccine efficacy studies, whilst simultaneously addressing fundamental questions on the immunological basis of susceptibility to iNTS disease. Several iNTS candidate vaccines are in development, but the epidemiology of iNTS disease is such that phase-III trials require a large financial and time commitment before efficacy readout is obtained. There are several instances where CHIM studies have accelerated vaccine candidates through to licensure, in particular for enteric pathogens such as *Vibrio cholerae* and *Salmonella* Typhi. We contend that a safe, reproducible, and well-designed CHIM – with clinically meaningful endpoints – will allow the field to build upon insights from animal-models and could have an important role in accelerating vaccine development for iNTS.

## Data Availability

No data are associated with this article.
