# Supplementary material for: The Challenge Non-Typhoidal Salmonella (CHANTS) Consortium: Development of a non-typhoidal Salmonella controlled human infection model: Report from a consultation group workshop, 05 July 2022, London, UK
Source: Wellcome Open Res. 2023 Sep 21;8:111. Originally published 2023 Mar 6. [Version 2] doi: 10.12688/wellcomeopenres.19012.2 (PMC10558987; doi:10.12688/wellcomeopenres.19012.2)
Supplement: Peer review history

# Reviewer report: Reviewer response for version 2

**DOI:** 10.21956/wellcomeopenres.22186.r67419

**Published:** 2023-10-06

**Authors:** Lisa E Emerson

**Affiliations:** 1 Emory Vaccine Center, Emory University, Atlanta, Georgia, USA

No further comments. Authors have included requested revisions.

Does the article adequately reference differing views and opinions?

Yes

Are all factual statements correct, and are statements and arguments made adequately supported by citations?

Yes

Is the Open Letter written in accessible language?

Yes

Where applicable, are recommendations and next steps explained clearly for others to follow?

Yes

Is the rationale for the Open Letter provided in sufficient detail?

Yes

Reviewer Expertise:

Vaccine development, Salmonella, extracellular vesicles

I confirm that I have read this submission and believe that I have an appropriate level of expertise to confirm that it is of an acceptable scientific standard.

# Reviewer report: Reviewer response for version 1

**DOI:** 10.21956/wellcomeopenres.21078.r63978

**Published:** 2023-08-25

**Authors:** Lisa E Emerson

**Affiliations:** 1 Emory Vaccine Center, Emory University, Atlanta, Georgia, USA

This article is an open letter detailing the conclusions of a group workshop regarding the development of a NTS vaccine. Several key challenges are made clear, including the host, ethical, and pathogenesis factors. The need for a NTS vaccine is made clear; however, what is not clear is the focus on NTS vs iNTS. In the safety and ethics section, it is suggested that the vaccine be tested against the ability to protect against invasive disease; however, the reasons why this is preferred to any NTS clinical symptoms were not clear. Since the host and bacterial factors that cause an NTS infection to become invasive are not well known, a serious barrier exists to the development of this CHIM. Additionally, over 2600 serovars of NTS exists, but the article does not make that clear.

 Of note, the selected challenge strain of ST313 is an excellent choice for creating a iNTS vaccine and the rationale written in the article is excellent.

 I recommend the acceptance of the article given that the authors revise the letter to clearly state 1) why protection against iNTS is preferred vs all NTS infections 2) the serovars the developed vaccine would protect against 3) the distinction between NTS, iNTS, and dNTS

Does the article adequately reference differing views and opinions?

Yes

Are all factual statements correct, and are statements and arguments made adequately supported by citations?

Yes

Is the Open Letter written in accessible language?

Yes

Where applicable, are recommendations and next steps explained clearly for others to follow?

Yes

Is the rationale for the Open Letter provided in sufficient detail?

Yes

Reviewer Expertise:

Vaccine development, Salmonella, extracellular vesicles

I confirm that I have read this submission and believe that I have an appropriate level of expertise to confirm that it is of an acceptable scientific standard, however I have significant reservations, as outlined above.

# Response

**Published:** 2023-09-07

**Authors:** Christopher Smith

**Affiliations:** Imperial College London, UK

Reviewer 2 Thank you for your comprehensive review and feedback. 

**"This article is an open letter detailing the conclusions of a group workshop regarding the development of a NTS vaccine. Several key challenges are made clear, including the host, ethical, and pathogenesis factors. The need for a NTS vaccine is made clear; however, what is not clear is the focus on NTS vs iNTS. In the safety and ethics section, it is suggested that the vaccine be tested against the ability to protect against invasive disease; however, the reasons why this is preferred to any NTS clinical symptoms were not clear. Since the host and bacterial factors that cause an NTS infection to become invasive are not well known, a serious barrier exists to the development of this CHIM."**

 Thank you. The primary focus of this meeting was to discuss the potential merits of developing a NTS CHIM, and the possible applications such a model could have in supporting candidate vaccine evaluation and licensure. Development of these vaccine candidates is undertaken by other stakeholders and is not within the remit of the CHANTS consortium. Vaccines currently in early-stage development are reviewed in the ‘NTS vaccine landscape’ section. Candidates utilise differing vaccine platforms and technologies; however, all are targeted at providing protection against *S. *Typhimurium and/or *S. *Enteritidis – serovars which are known to account for >90% of iNTS disease causing isolates. The ideal vaccine candidate would offer effective protection against both iNTS and dNTS. However, in these early stages, the priority is to develop vaccines which offer effective protection against invasive *Salmonella *disease. The rationale for this is highlighted in the stark differences in morbidity and mortality observed between iNTS and dNTS (see epidemiology figures in Introduction, paragraph 2). Furthermore, increasing multi-drug resistance amongst iNTS disease causing serovars (e.g., ST313 D23580) highlights the urgent need for effective vaccination strategies in endemic settings to avert further expansion of antimicrobial resistance. It is anticipated that successful candidates could be in bivalent formulations, or trivalent, providing coverage for both NTS and typhoidal serovars in relevant settings.  We wholly agree our understanding of the bacterial and host factors leading to differential clinical phenotypes (iNTS vs. dNTS) is incomplete. We aim to gain a deeper understanding of these factors through this first-in-human NTS CHIM, and this is a primary rationale for the study.

**"Additionally, over 2600 serovars of NTS exists, but the article does not make that clear."**  

 Thank you, this has been clarified in the introductory section.

**"Of note, the selected challenge strain of ST313 is an excellent choice for creating a iNTS vaccine and the rationale written in the article is excellent.** **I recommend the acceptance of the article given that the authors revise the letter to clearly state 1) why protection against iNTS is preferred vs all NTS infections 2) the serovars the developed vaccine would protect against 3) the distinction between NTS, iNTS, and dNTS."**

 Thank you. These clarifications have been highlighted in response to comments above and in the main body of the manuscript.

# Reviewer report: Reviewer response for version 1

**DOI:** 10.21956/wellcomeopenres.21078.r55591

**Published:** 2023-04-05

**Authors:** Joshua Osowicki

**Affiliations:** 1 Murdoch Children's Research Institute, Melbourne, VIC, Australia; 2 Department of Paediatrics, The University of Melbourne, Melbourne, VIC, Australia; 3 Infectious Diseases Unit, Royal Children's Hospital Melbourne, Melbourne, VIC, Australia

Thank you for the opportunity to review this Open Letter reporting on a 2022 CHANTS consortium workshop discussing a range of topics relevant to development of a non-typhoidal *Salmonella *(NTS) controlled human infection model (CHIM).

 The authors and workshop participants are well-qualified to have pursued this initiative and their broad expertise adds weight to the discussion described in the report and its recommendations.

 The report itself is a mature and thoughtful work, addressing contentious questions and areas of uncertainty with the even-handed seriousness that has become characteristic of modern investigator-initiated human challenge research (a standard set by a number of the authors and attendees at the workshop).

 At the outset, there is a familiar tension here, as for many CHIM initiatives, in balancing the experimental aims to address unanswered questions related to NTS infection and disease in humans with the vision of the CHIM as a safe and robust clinical trial platform. Again, I think the authors have done a good job here, making the case that an NTS CHIM meets a “safe enough and useful enough” threshold to pursue further, looking forward to the use case for a successful CHIM without pre-judging the outcomes of the initial efforts to establish the CHIM.

 The focus on study endpoints was strong, reminiscent of the 2018 publication by Chad Porter and colleagues regarding clinical endpoints in the Shigella CHIM (PMID: 29590182). The point that CHIM endpoints must be selected with specific consideration of vaccine indications and endpoints in later phase clinical trials was well made. Similar points are made in the Discussion section of the typhoid CHIM trial that evaluated the Vi-TT vaccine (Celina Jin et al, PMID: 28965718) and the linked editorial commentary (Feasey & Levine, PMID: 28965714).

 As with most well-written meeting reports, there are few targets here for a specific critique. As the authors describe, there will be important decisions in developing the final protocol for the first NTS CHIM study. The devilish details of the final protocol may inspire more specific criticisms.

 The meeting report outlines a cautious general approach, for an inpatient trial in extensively screened volunteers (including with abdominal ultrasound) with a relatively low threshold for antibiotic therapy and a relatively stringent requirement for microbiological eradication afterwards.

 The risk assessment table is excellent and a model for other CHIM investigators.

 I’m not certain that clinical examination can truly ‘exclude valvular heart disease’ given the significant subclinical fraction revealed by screening echocardiography studies (e.g. for rheumatic heart disease). I’m unconvinced that there’s a specific need for further screening on this front though, if all other ‘healthy adult’ requirements are met. Somewhat similar, I wonder what is the prevalence of subclinical aortic and biliary tract pathology in otherwise healthy young adults? That said, so long as a standardised approach is taken to performing and reporting the abdominal ultrasounds, and members of the clinical trial team are trained to complete these targeted ultrasounds, they may not be too onerous, too expensive, and won’t exclude too many otherwise eligible volunteers.

 The term ‘double-blind randomisation’ will eventually need to be defined in more detail. Who exactly will be blinded? Presumably only the volunteers, the clinical trials facility staff, and those charged with assessing endpoints? Too much blinding too early in the development of a CHIM may be overly restrictive and prevent the almost inevitable flexible adjustments the protocol will require as early lessons emerge.

 I understand the choice to exclude volunteers previously vaccinated against typhoid from the initial trial. Although resource constraints may preclude challenging some of these volunteers at a dose level that achieves a high attack rate in unvaccinated volunteers, I do wonder if samples from these participants could make a meaningful contribution to the immunological work to follow the clinical trial. Given the investments required to launch a CHIM study, this idea may not happen at all if it doesn’t happen early and in parallel to another study.

 The final reference regarding systems serology introduces these methods in relation to HIV research. The same others (Galit Alter et al) have since published systems serology investigations specifically related to CHIM studies (e.g. typhoid, shigella) that would seem to be even more appropriate references here?

 I know that these authors have a strong record of work with clinicians and investigators in low- and middle-income countries. As they point out, the global burden of NTS disease is concentrated in LMICs and especially in Africa. However, there is limited representation from LMICs including African countries (besides Prof. Gordon) in the author group for this open letter and the workshop attendees. I do trust the consortium has plans to boost its engagement in this regard. Nonetheless, it does bear mentioning. I do note the authors have made a general comment in the conclusion regarding plans for stakeholder engagement.

 The rationale for this Open Letter is clear: to introduce the CHANTS consortium and its mission to a broader audience, describing the rationale for developing an NTS CHIM and the seriousness with which the consortium is considering the safety of prospective participants and how to design the model in a way that will boost its contribution to accelerating NTS vaccine development. It is never possible to predict 100% of all impacts that will flow from the successful establishment of a new CHIM. The contributors to this meeting have made a strong case that a CHIM can be pursued with sensible safety guardrails (safe enough) and that it is a worthwhile objective that is more likely than not to meaningfully contribute to NTS science and vaccine development beyond what has been achieved to date with in vitro studies, animal models, and studies of naturally infected humans (useful enough). I look forward to news of the study protocol and the early findings that emerge from a new NTS CHIM.

Does the article adequately reference differing views and opinions?

Yes

Are all factual statements correct, and are statements and arguments made adequately supported by citations?

Yes

Is the Open Letter written in accessible language?

Yes

Where applicable, are recommendations and next steps explained clearly for others to follow?

Yes

Is the rationale for the Open Letter provided in sufficient detail?

Yes

Reviewer Expertise:

I am a paediatric infectious diseases physician and human infectious challenge researcher. In my PhD I led the CHIVAS-M75 study to establish a Streptococcus pyogenes pharyngitis CHIM in healthy adult volunteers. I am now undertaking S. pyogenes challenge trials to evaluate vaccines and accelerate development of improved long-acting penicillin formulations (primarily for secondary prevention of rheumatic heart disease). I am also involved in efforts towards developing new human models of bacterial and mycobacterial skin infections and sexually-transmitted infections.

I confirm that I have read this submission and believe that I have an appropriate level of expertise to confirm that it is of an acceptable scientific standard.

# Response

**Published:** 2023-09-07

**Authors:** Christopher Smith

**Affiliations:** Imperial College London, UK

Reviewer 1 Thank you for your comprehensive review and feedback. 

**"I’m not certain that clinical examination can truly ‘exclude valvular heart disease’ given the significant subclinical fraction revealed by screening echocardiography studies (e.g. for rheumatic heart disease). I’m unconvinced that there’s a specific need for further screening on this front though, if all other ‘healthy adult’ requirements are met. Somewhat similar, I wonder what is the prevalence of subclinical aortic and biliary tract pathology in otherwise healthy young adults? That said, so long as a standardised approach is taken to performing and reporting the abdominal ultrasounds, and members of the clinical trial team are trained to complete these targeted ultrasounds, they may not be too onerous, too expensive, and won’t exclude too many otherwise eligible volunteers."**

 Thank you. We wholly agree on points raised regarding potential subclinical aortic, valvular, and biliary tract disease. Our intended participant cohort will be comprised of healthy adults (aged 18-50) without chronic disease or risk factors for severe Salmonellosis. Screening protocols are designed to select such individuals through comprehensive medical history, physical examination, and review of primary care records. We recognise the pre-test probability of subclinical biliary or aortic disease in this cohort is very low. However, we have agreed on abdominal ultrasonography with standardised assessment and reporting protocols as an appropriate measure to exclude these specific risk factors for complicated infection. Abdominal USS is accessible, non-invasive, and can be performed and reported in a timely manner. Transthoracic echocardiography was carefully considered but felt unnecessary due to burden of imaging and reporting times – again, in the context of a low pre-test probability for subclinical valvular disease. Furthermore, *Salmonella *endocarditis is thought to represent a less likely complication even amongst the small proportion who may develop bacteraemia. Timely antibiotic therapy will be commenced immediately upon development of sustained fever and/or bacteraemia, significantly reducing the probability of such complications developing.

**"The term ‘double-blind randomisation’ will eventually need to be defined in more detail. Who exactly will be blinded? Presumably only the volunteers, the clinical trials facility staff, and those charged with assessing endpoints? Too much blinding too early in the development of a CHIM may be overly restrictive and prevent the almost inevitable flexible adjustments the protocol will require as early lessons emerge."**

 Thank you. As suggested, blinding will be applied to participants, clinical study staff delivering direct patient care, and the same clinical team members responsible for assessment of clinical endpoints. Unblinding will occur at study conclusion, or as deemed necessary by the Data Safety and Monitoring Committee in response to safety thresholds.  

**"I understand the choice to exclude volunteers previously vaccinated against typhoid from the initial trial. Although resource constraints may preclude challenging some of these volunteers at a dose level that achieves a high attack rate in unvaccinated volunteers, I do wonder if samples from these participants could make a meaningful contribution to the immunological work to follow the clinical trial. Given the investments required to launch a CHIM study, this idea may not happen at all if it doesn’t happen early and in parallel to another study."**

 Thank you. Participants will only be excluded if they have received prior vaccination with oral typhoid Ty21a vaccine (or confirmed prior *Salmonella *infection). As Ty21a is a live attenuated vaccine, this would be considered prior ‘oral challenge’ with a clinically relevant *Salmonella *serovar. No other prior formulation of typhoid vaccination would preclude participation. Immunological assays will be applied following the clinical study on samples obtained from all participants, irrespective of attack rate.

**"The final reference regarding systems serology introduces these methods in relation to HIV research. The same others (Galit Alter et al) have since published systems serology investigations specifically related to CHIM studies (e.g. typhoid, shigella) that would seem to be even more appropriate references here?"**

 Thank you. The final references have been updated to reflect your appropriate suggestions.

**"I know that these authors have a strong record of work with clinicians and investigators in low- and middle-income countries. As they point out, the global burden of NTS disease is concentrated in LMICs and especially in Africa. However, there is limited representation from LMICs including African countries (besides Prof. Gordon) in the author group for this open letter and the workshop attendees. I do trust the consortium has plans to boost its engagement in this regard. Nonetheless, it does bear mentioning. I do note the authors have made a general comment in the conclusion regarding plans for stakeholder engagement."**

 Thank you for this important comment. As suggested, we aim to maximise engagement with key stakeholders from LMICs, where the greatest burden of iNTS disease is concentrated. Members from academic and research institutions in sub-Saharan Africa have been consulted throughout the protocol development stages of this study, and also form central roles within our Data Safety and Monitoring Committee. Future applications and use case of the model will involve ongoing collaboration with representatives from SSA, including potential transfer of the model to an endemic setting, and in planning future vaccine efficacy studies.
